# New Definition of Transplant Tourism

**Published:** 2017-02-01

**Authors:** B. Broumand, R. F. Saidi

**Affiliations:** 1 *Emeritus Professor of Medicine, Pars Advanced and Minimally Invasive Manners Research Center, Pars Hospital, Iran University of Medical Sciences, Tehran, Iran*; 2 *Assistant Professor of Surgery, Division of Organ Transplantation, Department of Surgery, Alpert Medical School of Brown University, 593 Eddy Street, Providence, RI 02903, USA*

Kidney transplantation is the treatment of choice for patients with end-stage renal disease (ESRD). Improvement in surgical care, tissue typing, and immunosuppression management has dramatically improve the outcome of kidney transplant recipients.

With all of these achievements, organ shortage globally is one the challenges facing transplantation. One step for meeting this need is utilization of living unrelated from volunteer friends and emotionally related (husband or wife), the so-called “altruistic donation.” Globally, kidney transplantation from unrelated-living donors considered the major donor source. The main reason for expansion of this source was the need of poor people for money so their kidney was used as a commodity. At this point, the influence of sociocultural factors on organ donation and transplantation is a major concern. There was not enough volunteer to donate the kidney so the recipients had to pay the donors, the vendors and brokers for the kidney. The demand for organ transplants in industrial countries is rising much faster than the supply of organs donated through traditional means. In response, a small but growing number of the world’s poor people are offering their body parts for transaction, and kidneys are the most commonly purchased organs [1].

One hope was transplantation from deceased donors, but this solution did not solve all of the problems. In some countries the concept of brain death was not accepted [2]. In other countries, health care professionals did not believe to transplant deceased donor kidney. For instance, in a recent paper by Qsama Al and colleagues, it is mentioned that “more than half of the physicians (59.7%) and technicians (57.4%) assumed that organs can be bought and sold in the country” [3]. This approach indeed exists in most of the Middle East countries and was the basis for transplant commercialism and tourism.

According to WHO, “transplant tourism” refers to patients travelling across the borders to be transplanted elsewhere [4]. People tend to travel for transplantation, either because it is not available in their home country, such as Tajikistan and Azerbaijan, or if the facilities are adequate in their home land, there are not enough organs available.

Transplant tourism takes place in two different situations: 1) in very well developed countries with long waiting list, and 2) in underdeveloped countries with no prohibitory regulations for buying and selling the kidney but the people are indigent and have to make money by selling their organs.

After Istanbul declaration, commercialism for transplant has become harder (and in some places impossible), and the need to find an organ has led to an alternate solution, mainly through the temptation into organ trafficking and transplant tourism.

The most common way to trade organs across national borders is via potential recipients who travel abroad to purchase kidney and undergo organ transplantation, commonly referred to as “transplant tourism.”

The concept of transplant tourism is confusing and vague. According to Shimazono, transplant tourism refers to “overseas transplantation in which a patient obtains an organ through the organ trade or other means the organ trade may take other forms as well.” For example, Shimazono reported that live donors have been brought from the Republic of Moldova to the USA or from Nepal to India [4]. In other cases, both recipients and donors from different countries move to a third country that contravenes the regulatory framework of their countries of origin [4]. The advantages and disadvantages of transplant tourism have been addressed in another paper [5]. So to bypass all of obstacles, the solution has been going outside of national border to the countries with available excuse to buy an organ from deceased donor.

In many countries law prohibits selling organs from natives to foreigners [6]. Lately, a very clever trick discovered to bypass this barrier. This is bringing some healthy people from outside the border inside and use their organs with the excuse of an accord [7].

Since January 2014, with the excuse of support of Istanbul declaration and combat of transplant commercialism, the Doha donation accord was suggested by Hamad Medical Corporation, Doha, Qatar in Transplantation [16], the authors explain “…Qatar is notable for the fact that organs for transplantation are equitably allocated within Qatar to suitable Qataris and expatriate patients residing in Qatar alike, without regard to citizenship status.”

According to Hanan Alkuwari, *et. al.*, from Hamad Medical Corporation, in 2014, due to lack of donors in Qatar, many patients with end-stage renal disease sought commercial transplantation, but returned to their country with high rates of post-operative complications (68%) and early post-operative mortality (12%) [8]. Even if we accept that the expatriate has the same privilege for allocation of deceased organ as native of Qatar, the country is still using transplant tourism from 60% of expatriate [9]. To support this concern, I refer readers to an article published in the guardian in 2013 [9]. This article is about the condition of migrant workers in Qatar, the so-called “expatriates.” It tells a sad story that may not be known to many. In reality, these expatriates are often very poor non-immigrant foreigners, paid miserly wages and are often the victims of accidents in risky jobs such as construction. They have a sad fate in Qatar.

The main concern is that the Doha Donation Accord in the present form, will lead to another form of transplant tourism, from expatriate donors to Qatari citizens or citizens of other Persian Gulf nations. If both sides of the equation were citizens of the same nation, there would be little problem. However, when the recipients of organs are almost exclusively Qataris or citizens of rich Persian Gulf nations and the donors are almost exclusively expatriates who are in Qatar as cheap labor, the story would be different.

I think the term “regardless of their nationalities,” used in the article, will open the way for organs from expatriates for the use by the recipients from rich countries, which in the article are given the label of those “who rank high in human development.” In our own view, this latter term would be a justification for organ tourism.

The authors said that “Because more than 80% of the potential donors are expatriate workers living alone in Qatar with their families abroad, the multicultural and metalinguistic team of donation coordinators and social workers develops a supportive relationship with the donor family independent of the intensive care unit team.” It seems that the donated organ can come from a citizen of one nationality and the recipient can be from another nationality; to us this means organ tourism. Also notable is that many of the donors have travelled to Qatar to be able to send money back home to their families, which would suggest that their families are likely too poor to travel to Qatar to claim the bodies of their loved ones. This assumes that the poverty-stricken family back home is accessible so they can be told in a timely fashion what has happened to their loved one.

The authors also claimed that “Qatar is notable for the fact that organs for transplantation are equitably allocated within Qatar to suitable Qataris and expatriate patients residing in Qatar alike, without regard to citizenship status.” The real life translation of this is likely to be that this phrase “without regard to citizenship status” will be used to justify that the organ from a native of Somali or a Nepal, for instance, can be transplanted to a recipient from Saudi Arabia or Qatar, and this is in fact a legitimization of organ tourism.

In the last three lines of page 3 of the article, it reads “Provisions of the DDA are accessible only by related live donors and recipients residing in Qatar (Qatari and expatriates), and not by visitors.” We agree that the visitors should be prevented from coming into the country to purchase organs but why should the organs from a Nepalese expatriate be shipped to Saudi Arabia (according to the authors)? This would indeed make it transplant tourism. In other words, while the visitors cannot be the recipients, the organ could be shipped to Saudi Arabia. 

Considering these facts and our previous experience with “organ brokers” in Iran, Iraq, Egypt, Pakistan and other countries, we know that unfortunately the brokers will find a way to exploit poor people in Qatar [10]. We are sure if Qatar establishes a registry for deceased organ procurement, it could be uncovered that almost 100% of procured organs are from expatriates.
